# Caregiver Burdens Associated With Treatment-Resistant Schizophrenia: A Quantitative Caregiver Survey of Experiences, Attitudes, and Perceptions

**DOI:** 10.3389/fpsyt.2019.00584

**Published:** 2019-09-06

**Authors:** Dawn I. Velligan, Cecilia Brain, Laëtitia Bouérat Duvold, Ofer Agid

**Affiliations:** ^1^Department of Psychiatry, University of Texas Health Science Center, San Antonio, TX, United States; ^2^Medical Affairs, H. Lundbeck A/S, Valby, Denmark; ^3^Centre for Addiction and Mental Health (CAMH), University of Toronto, Toronto, ON, Canada

**Keywords:** treatment-resistant schizophrenia, positive symptoms, caregiver, burden, survey

## Abstract

**Background:** Previous qualitative studies indicate high caregiver burden associated with providing care for people with treatment-resistant schizophrenia (TRS). In this study, we report the first quantitative data to characterize the burdens of caring for a person living with TRS. To better understand the potential added burdens of persistent positive symptoms, we compared the self-reported burdens of caregiving for people living with TRS *versus* caregiving for those whose positive schizophrenia symptoms respond to treatment (comparator group).

**Methods:** Non-professional adult caregivers providing ≥20 h/week of care to individuals with schizophrenia completed an online survey. Allocation to the TRS or comparator groups was based on caregiver report. TRS was defined as failure of ≥2 separate antipsychotics and at least moderate severity in two of four persistent core positive symptoms despite medication adherence. Care recipients in the comparator schizophrenia group had no clinically significant positive symptoms.

**Results:** One hundred seventy seven caregivers (n = 100 TRS group, n = 77 comparator group) completed the online survey. Caregivers in both groups reported high levels of every day involvement in most aspects of daily life, including assistance with basic tasks, housekeeping, and in providing emotional support. There were no significant differences between groups on overall social life or health. However, caregivers of people living with TRS were significantly more likely to be experiencing stress (76% *vs*. 53%) and anxiety (58% *vs*. 43%). Relevant differences between caregiver groups were also noted for mean number of hours spent per week on direct care (TRS group *vs*. comparator group: 61.1 h/week *vs*. 39.7 h/week, respectively) and time spent “on call” (162.8 h/week *vs*. 121.6 h/week). Amongst the caregivers in the TRS group, correlation analyses revealed moderate positive correlations between the reported burden of individual persistent positive symptoms with overall caregiver burden.

**Conclusion:** Our findings show that caring for a person living with TRS places considerable burden on caregivers’ lives, with the severity of the disease (and especially severity of positive symptoms) driving further burden, as highlighted by a number of quantitative differences between the TRS and comparator groups.

## Introduction

It is currently estimated that up to a third of people living with schizophrenia experience persistent psychotic symptoms despite treatment with antipsychotics (1, 2). Failure of two or more different antipsychotic treatments of adequate dose and duration (6–8 weeks of antipsychotic therapy) meets the clinical guideline criteria for treatment-resistant schizophrenia (TRS) (1–3). While the definition of TRS has progressed from its original description in 1988 (4) to incorporate negative and cognitive symptoms (1–3), persistent positive symptoms remain central as they cause a high patient and caregiver burden (5) and constitute the main target of antipsychotics (6, 7). The only pharmacotherapy currently recommended for TRS is clozapine, which treatment guidelines recommend after two treatment failures (8–10).

In recent years, there has been growing agreement that TRS is categorically distinct from treatment-responsive schizophrenia (11). TRS can be present from early on in the disease course, or can develop later after multiple relapses (8) or other reasons (3, 9, 10). Various hypotheses have been proposed for the causes of TRS, with some suggesting a non-dopaminergic basis and others proposing that TRS may be caused by the development of dopamine D2 receptor supersensitivity with the long-term administration of antipsychotics (11–16). Regardless of etiology, the management of TRS poses a difficult mental health care challenge that is different to treatment-responsive schizophrenia. People living with TRS do not experience sustained symptom relief and, at the same time, often have the most severe disease-related disability, cognitive dysfunction, and worse community functioning (17–19). From the healthcare and economic perspective, TRS is associated with higher rates of hospitalization, increased health resource utilization costs, and higher unemployment rates than in the broader schizophrenia population. TRS usually causes a significant impact on all aspects of short- and long-term outcome (17, 18).

Caregivers often experience significant disease burden along with their care recipients (20). Previous research exploring the caregiver experience in the broader schizophrenia population has demonstrated considerable objective and subjective burdens of providing informal care (19–22), especially on the caregivers’ own mental health and daily functioning (23). Focusing on TRS, we recently reported the results of a qualitative study conducted with caregiver focus groups showing that persistent positive symptoms cause significant perceived burden, feelings of being overwhelmed as well as having no relief, and substantial negative impacts on caregivers’ emotional and physical health (5). In that study, caregivers reported an average of 36.8 h/week providing direct (face to face) care, with most (78%) reporting being *“on call 24/7”* regardless of where they were or what they were doing.

It has been suggested that—due to the relentless nature of uncontrolled positive symptoms—the burden of caring for someone with TRS may be higher than caring for someone with responsive symptoms (16). In this quantitative survey, we aimed to further characterize the different burdens of caring for people with TRS, and to compare the impact of these burdens with that of caring for a person whose positive symptoms of schizophrenia respond to treatment.

## Methods

### Recruitment and Setting

The study was conducted in compliance with relevant codes of conduct including the Market Research Society guidelines, Council of American Survey Research Organizations (CASRO, now known as Intellus) guidelines and data protection legislation. As noted in the exemption criteria outlined by The Department of Health and Human Services (see https://www.hhs.gov/ohrp/regulations-and-policy/regulations/45-cfr-46/index.html#46.101(b)), this market research study does not require Clinical Research Ethics Committee or Independent Review Board (IRB) approval. As such, Pearl IRB waived the requirement for ethical approval for this study. All respondents consented to participate in the survey. They were made aware that the research was on behalf of a pharmaceutical company interested in different mental health conditions.

Caregivers were recruited and screened over the telephone by specialist recruiters to meet quotas by USA region (in line with the US 2017 Census, we used a ratio of 1:1:1:2 for North East : Midwest:West : South, respectively) and by relation to the person living with schizophrenia (ratio of 3:2:2:1:2 for parent:sibling:spouse:adult child:other, respectively). Caregivers were identified by the specialist recruiters *via* established links with support and advocacy groups, referrals (e.g., *via* nurses and psychiatrists) and advertisement in appropriate spaces and publications. An initial target was set to recruit 100 caregivers of persons with TRS and 75 caregivers of persons whose positive symptoms of schizophrenia respond to treatment (the comparator group). Caregivers who met inclusion criteria were sent a link to the confidential online survey.

### Recruitment Criteria

Adult caregivers (both groups) were eligible for inclusion in the study if they were an unpaid caregiver (aged ≥18 years) for an adult individual diagnosed with schizophrenia (time since diagnosis ≥1 year and aged ≥19 years old). Caregivers had to have cared for the recipient for ≥1 year, and they should spend ≥20 h/typical week providing care, of which 4 h must be direct care.

Care recipients had to currently be on antipsychotic medication and adherent, defined here as taking their past and current medications as prescribed (≥80% of the time) as assessed by the caregiver. Caregivers providing care to people *currently* taking clozapine were not eligible for the study. The main reason for this exclusion was that few individuals with schizophrenia are prescribed clozapine, and caregivers of individuals receiving clozapine might not represent the real-world experiences of those caring for individuals with TRS. Despite strong meta-analytic evidence that clozapine is significantly better at treating the positive symptoms of TRS than first-generation antipsychotics and some second-generation antipsychotics (6, 24), prescription patterns data show the use of clozapine is underused (25). However, *prior* clozapine use was permitted provided it was not discontinued due to inefficacy. The care recipient could also have caregiver report of co-morbid bipolar-/manic-depressive disorder or other schizophreniform disorders as long as schizophrenia was the most current diagnosis.

Allocation to the TRS or comparator groups was based on caregiver report only (i.e., there was no cross check with medical records). TRS was operationalized as failure of ≥2 separate antipsychotics (taken as prescribed for ≥6 weeks, including ≥1 atypical) and at least moderate severity in two of four persistent core positive symptoms (hallucinations, delusions, disorganized behavior and/or speech, suspiciousness/persecution) despite medication adherence, as reported by the caregiver. Moderate symptoms were described in the survey *“as occurring on more than half the days; somewhat bothered by schizophrenia symptoms; some pressure to respond to delusional beliefs; speech is often difficult to follow.”* For the comparator group, care recipients had to have no more than “mild” positive symptoms on their current treatment (without being entirely asymptomatic for positive symptoms). They may also have experienced at least moderate severity for one of four core positive symptoms while on a previous treatment, but this had to be currently reported as diminished severity for that symptom. There were no restrictions based on the negative or cognitive symptoms of schizophrenia.

### Survey Design

A pilot questionnaire was developed by the authors in collaboration with Healthcare Research Worldwide (HRW) and was tested in interviews with 10 caregivers (n = 7 caring for someone with TRS and n = 3 caring for someone whose positive symptoms of schizophrenia respond to treatment) between 30th April 2018 and 11th May 2018. Questions were largely developed specifically for use in this study and were based on findings from the prior qualitative study (5). Also imbedded within the survey was the validated Schizophrenia Caregiver Questionnaire (SCQ; rated on a scale of 0–100), which assesses the humanistic impact, exhaustion with caregiver role, lack of support, care recipient dependence, worries for the care recipient, perception of care provided, finance, and overall difficulty of caring for a person living with schizophrenia (26, 27). For the testing phase, caregivers completed the survey online while on the telephone with a trained interviewer, using a screen sharing platform. The purpose of the pilot interviews was to test the survey for functionality, comprehension, and relevance of questions. Anything that was unclear was discussed during the interview, and the initial questionnaire was amended to improve clarity of language and improve available answer options.

The final survey comprised two main parts: a scripted screening questionnaire and an online survey. In the screening part of the survey, caregiver respondents answered questions to determine eligibility for the study. The care recipient’s symptoms of schizophrenia were assessed using predefined lists (positive symptoms: hallucinations, delusions, disorganized behavior and/or speech, suspiciousness/persecution; additional symptoms: agitation/hostility, cognitive impairment, difficulty in expressing emotions, poor rapport, and social withdrawal). Caregivers were provided with clear examples of each symptom and were asked to rate the severity of symptoms while on the current medication as well as for the prior two medications.

The online survey was designed to take no more than 30 min, but it could be saved and completed in more than one sitting. It included over 80 closed and open-ended questions (including the SCQ) to evaluate caregiver attitudes, experiences, and perceptions associated with caring for someone with schizophrenia. The actual number of questions per caregiver depended on the responses given. For some questions (including those related to stigmatization), caregivers were asked to rate their agreement with a series of statements on a scale of 0–10 (no agreement to full agreement). For questions regarding caregiver involvement with daily tasks, caregivers were asked to choose from “essential” (every day), “significant” (a few times/week), “limited” (a few days/month), or “none.”

### Data Analysis

Descriptive statistics (n, mean and SD or median and range) were used to summarize survey items collected in this study. Differences between the TRS and the comparator group on means for the previously validated SCQ section of the questionnaire were tested for using a multivariate analysis of variance (MANOVA). Between group differences on each of the other, exploratory questions were primarily tested for using a series of univariate T-tests, with no adjustment for multiplicity. Differences between groups in caregiver mental and physical health impacts were assessed using a Χ^2^ test. We used the Pearson product-moment correlation coefficient to estimate the relative contribution of each positive symptom to overall burden, where the percent of caregivers scoring 8–10 out of 10 (i.e., high burden) regarding the burden of each core symptom was tested against the average score on SCQ.

## Results

### Caregiver and Care Recipient Characteristics

A total of 177 caregivers of people living with schizophrenia (n = 100 TRS group, n = 77 comparator group) were recruited to complete the online survey. Key characteristics for caregivers and care recipients are provided in **Table 1** and reflect the respective recruitment criteria for the two groups. Participating caregivers were primarily white, females with a mean age of 50, taking care of primarily males with schizophrenia who were in their late 30s to early 40s (mean 39 years). The majority of participating caregivers had at least a college education, and half were parents of the care recipient. Compared to those in the comparator group, care recipients with TRS were slightly older with a longer time since diagnosis; they were also more likely to be single and unemployed.

**Table 1:** Caregiver and care recipient characteristics*.

Characteristic	TRS group (n = 100)	Comparator group (n = 77)
Caregiver characteristics		
Age, years (mean ± SD) Age categories, n (%) * 19–24* * 25–34* * 35–44* * 45–54* * 55–64* * 65+*	51 ± 12.7 1 (1) 9 (9) 20 (20) 23 (23) 32 (32) 15 (15)	48 ± 10.6 0 9 (11.7) 20 (26) 27 (35.1) 16 (20.8) 5 (6.5)
Sex, n (%) * Male* * Female*	18 (18) 82 (82)	19 (25) 58 (75)
Race, n (%) *White Caucasian* * African-American/Black* * Asian* * Hispanic/Latino* * Other*	69 (69) 12 (12) 1 (1) 12 (12) 7 (7)	60 (78) 6 (8) 2 (3) 11 (14) 1 (1)
Employment status, n (%) * Employed full time* * Employed part time* * In Education/studying* * Volunteering* * Retired/homemaker* * Unemployed* * Rather not say*	43 (43) 25 (25) 1 (1) 2 (2) 20 (20) 7 (7) 2 (2)	42 (55) 18 (23) 0 (0) 1 (1) 6 (8) 8 (10) 2 (3)
Education level, n (%) *High school graduate* * Some college* * College graduate (2/4 years)* * Graduate school* * Technical/vocational school* * I’d rather not say*	4 (4) 24 (24) 39 (39) 29 (29) 3 (3) 1 (1)	5 (7) 18 (23) 37 (48) 12 (16) 4 (5) 1 (1)
Caregiver relationship, n (%) *Parent* * Sibling* * Spouse/Partner* * Adult child* * Another family member* * Friend or other unpaid, non-professional caregiver*	51 (51) 19 (19) 10 (10) 14 (14) 2 (2) 4 (4)	37 (48) 5 (7) 18 (23) 11 (14) 1 (1) 5 (7)
Sole caregiver, n (%)	62 (62)	41 (53)
**Care recipient characteristics**		
Age, years (mean ± SD) (range)	40 ± 17.6 [18–65+]	37 ± 14.1 [18–65+]
Sex, n (%) *Male* * Female*	74 (74) 26 (26)	50 (65) 27 (35)
Marital status, n (%) *Single* * Married/long-term relationship* * Divorced* * Widowed*	74 (74) 13 (13) 6 (6) 7 (7)	42 (55) 28 (36) 7 (9) 0 (0)
Employment status, n (%) *Employed full time* * Employed part time* * In education/studying* * Volunteering* * Retired/homemaker* * Unemployed*	3 (3) 10 (10) 8 (8) 0 (0) 8 (8) 70 (70)	9 (12) 26 (34) 8 (10) 4 (5) 9 (12) 21 (27)
Living arrangement, n (%) *Alone* * With caregiver* * With family* * Sheltered home/supported living* * With friends* * No fixed address*	20 (20) 52 (52) 11 (11) 10 (10) 6 (6) 1 (1)	13 (17) 42 (55) 14 (18) 5 (7) 3 (4) 0 (0)
Time since diagnosis, years (mean ± SD)	11.7 ± 10.0	8.7 ± 7.8
Time with caregiver, years (mean ± SD) (range)	15 ± 11.8 [< 5–20+]	12 ± 10.2 [< 5–20+]
Time on current medication, years (mean ± SD)	5.1 ± 6.0	4.4 ± 4.6
Current medication regime, n (%) Monotherapy Combination therapy	78 (78.0) 22 (22.0)	58 (75.3) 19 (24.7)
Had previously tried clozapine and discontinued**	34 (34.0)	43 (55.8)

*All available data, we did not ask the same demographic questions for caregivers and care recipients. **Caregivers of people who discontinued clozapine due to inefficacy were not included in the study.

In accordance with the target inclusion criteria, caregivers of people living with TRS reported that the care recipients had a higher rate of moderate to severe positive symptoms than those in the comparator group (**Table 2**). Of note, caregivers in the comparator group reported that the care recipient had *previously* experienced moderate–severe positive symptoms (now resolved or slight/mild). Rates of previous moderate–severe symptoms in the comparator group were: hallucinations (63.6%), delusions (46.8%), disorganized speech (44.2%), and suspiciousness/persecution (41.6%). For the TRS group, almost half (46%) of caregivers reported that the care recipient had experienced persistent positive symptoms since they started treatment for schizophrenia, and their symptoms had never improved despite the different antipsychotic medications that they have taken as prescribed (median of five prior switches *vs*. three prior switches for the comparator group). A further 35% reported that their care recipients’ positive symptoms had initially improved but had come back within 5 years of diagnosis, and 4% reported that symptoms had initially improved but had returned after 5 years since diagnosis; the remaining 15% of caregivers were unsure. About a third (34%) of care recipients in the TRS group had previously received clozapine and had discontinued due to the need for monitoring/blood draws, side effects, and other reasons excluding lack of efficacy (exclusion criteria).

**Table 2:** Presence of persistent positive symptoms.

Caregiver’s perceived severity of current core symptoms, n (%)	TRS group (n = 100)					Comparator group (n = 77)				
	None	Slight	Mild	Moderate	Severe	None	Slight	Mild	Moderate	Severe
Hallucinations	4 (4)	14 (14)	29 (29)	32 (32)	21 (21)	12 (16)	36 (47)	29 (38)	0 (0)	0 (0)
Delusions	10 (10)	11 (11)	31 (31)	34 (34)	14 (14)	18 (23)	35 (46)	24 (31)	0 (0)	0 (0)
Disorganized behavior and/or speech	8 (8)	15 (15)	17 (17)	38 (38)	22 (22)	17 (22)	35 (46)	25 (33)	0 (0)	0 (0)
Suspiciousness/persecution	8 (8)	10 (10)	22 (22)	34 (34)	26 (26)	21 (27)	27 (35)	29 (38)	0 (0)	0 (0)
**Perceived frequency of current additional behaviors, n (%)**	**None**	**Rarely**	**Several days a month**	**More than half the days in a month**	**Nearly every day or everyday**	**None**	**Rarely**	**Several days a month**	**More than half the days in a month**	**Nearly every day or everyday**
Agitation/hostility	16 (16)	22 (22)	30 (30)	18 (18)	14 (14)	8 (10)	24 (31)	32 (42)	10 (13)	3 (4)
Cognitive impairment	3 (3)	18 (18)	17 (17)	30 (30)	32 (32)	2 (3)	28 (36)	19 (25)	12 (16)	16 (21)
Difficulty expressing emotions	8 (8)	10 (10)	28 (28)	30 (30)	24 (24)	5 (7)	18 (23)	19 (25)	23 (30)	12 (16)
Poor rapport	9 (9)	11 (11)	26 (26)	32 (32)	22 (22)	5 (7)	22 (29)	26 (34)	12 (16)	12 (16)
Social withdrawal	3 (3)	10 (10)	20 (20)	35 (35)	32 (32)	6 (8)	16 (21)	22 (29)	24 (31)	9 (12)

### Burden of Persistent Symptoms

**Figure 1:**
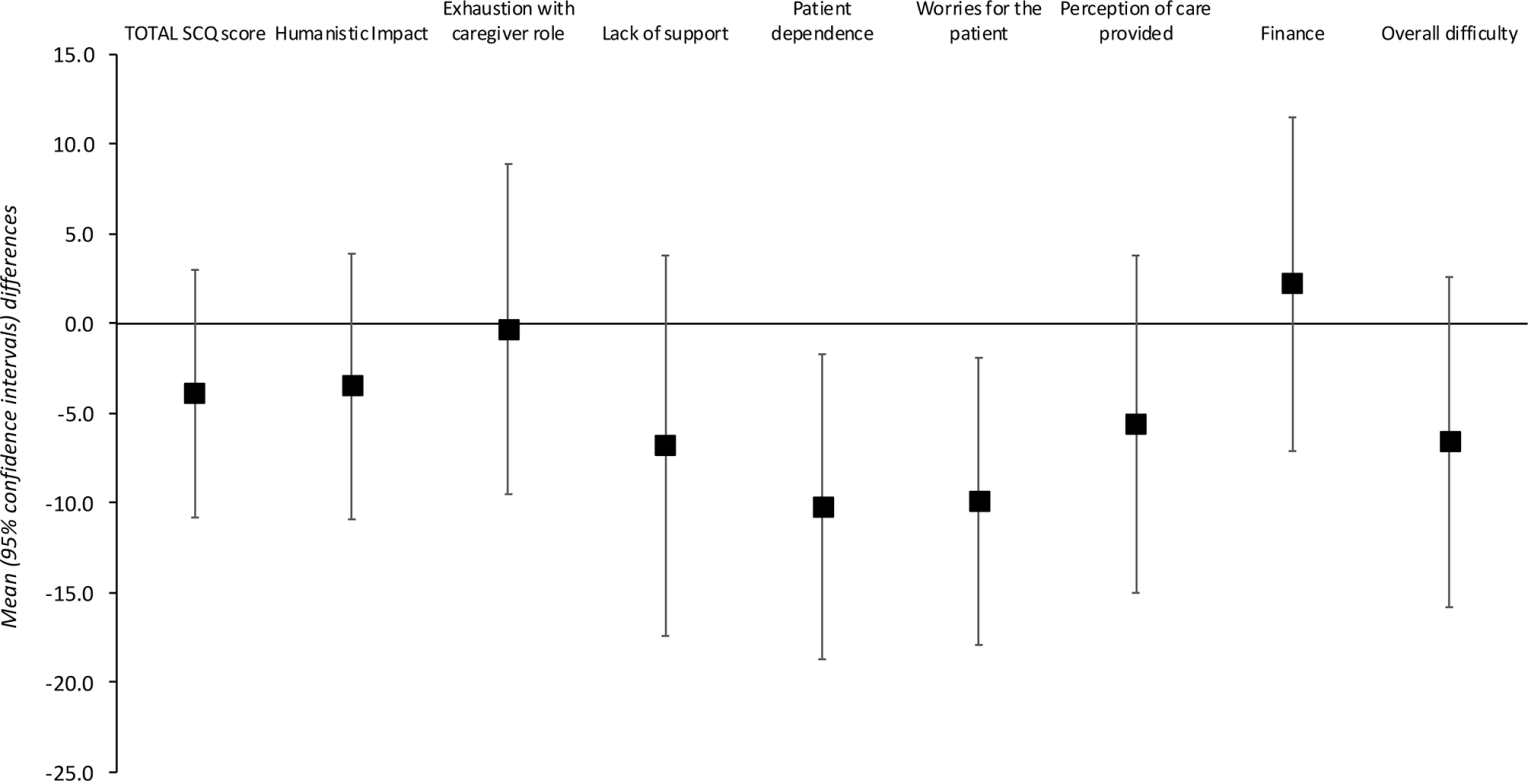
Schizophrenia Caregiver Questionnaire total and individual item scores.

The overall burden of caregiving for TRS was assessed with the SCQ; mean SCQ scores were 50.6 for the TRS group and 46.7 for the comparator group. Multivariate analyses confirmed a significant variance between groups, Wilks’ lamdba 0.891, *F (8, 168)* = 2.57, p = 0.01. On t-tests, group scores for the SCQ items of “acceptance of treatment” and “importance of medication” were significantly higher for the TRS *versus* comparator groups (p < 0.05) (**Figure 1**).

Amongst the caregivers in the TRS group, Pearson product-moment correlation analyses revealed moderate positive correlations (r >0.2 and <0.5) between the reported burden of all individual persistent symptoms with overall caregiver burden (SCQ total score). The strongest correlations were for the burdens of agitation/hostility (r = 0.45), suspiciousness/persecution (r = 0.43), and delusions (r = 0.41), and the overall correlation with the SCQ total score was 0.52. Similar analyses for the comparator group identified the burdens of poor rapport (r = 0.56), cognitive impairment (r = 0.52), and disorganized speech (r = 0.49) as the most strongly correlated with overall burden.

### Direct and Indirect Burden of Care

**Figure 2:**
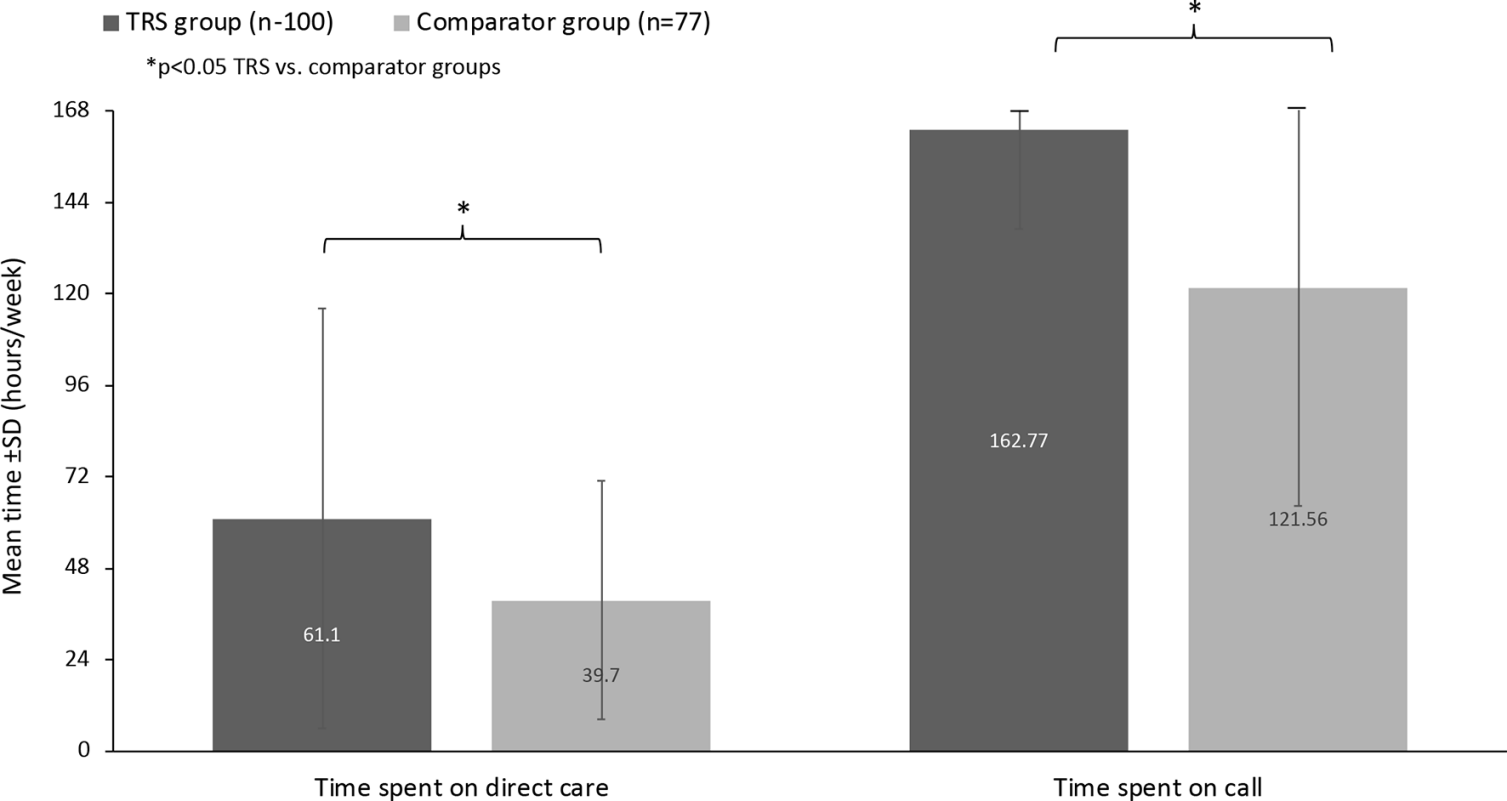
Overall caregiver involvement in daily tasks.

Caregivers of both groups reported high levels of “essential” (every day) involvement in a range of daily tasks (**Figure 2**). Differences between the TRS and comparator groups were noted for the percentage of caregivers reporting essential involvement in companionship and encouraging the care recipient to take care of their physical health (both p < 0.05).

**Figure 3:**
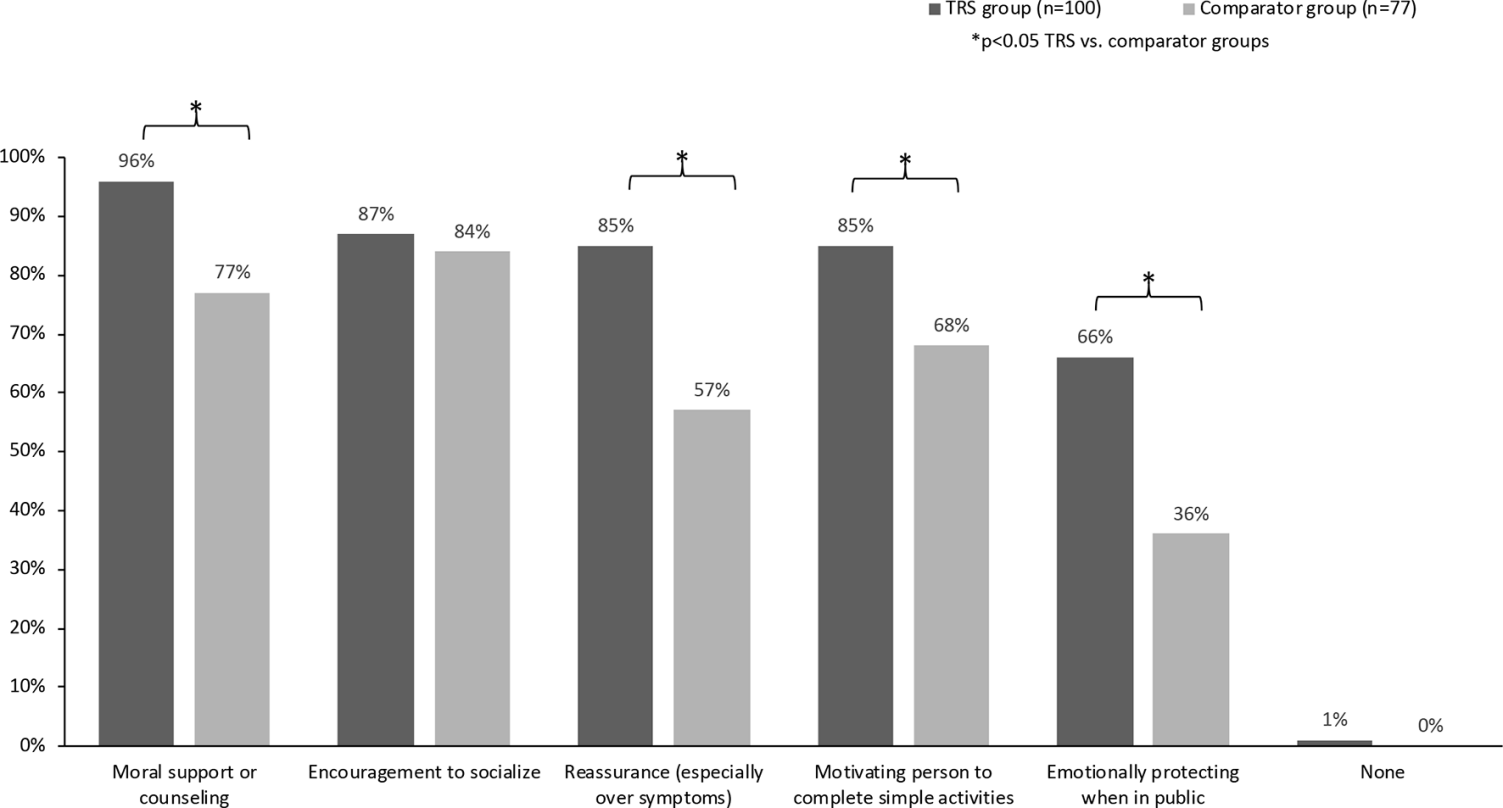
Time spent providing care (hours per week).

Relevant differences between caregiver groups were noted for hours spent per week on direct care and time spent “on call” (**Figure 3**). Overall, caregivers of persons living with TRS spent a mean ± SD of 61.1 ± 55.1 h/week providing direct care *versus* a mean of 39.7 ± 31.3 h/week for the comparator group. The mean difference between groups was −21.4 h/week (95% CI: −35.3, −7.6). Considering the amount of time spent “on call,” caregivers in the TRS group spent more time than those in the comparator group (162.8 ± 25.8 h/week *versus* 121.6 ± 57.2 h/week, respectively). The mean difference between groups was 41.2 h/week (95% CI: −53.9, 28.5).

### Burden of Emotional Support

**Figure 4:**
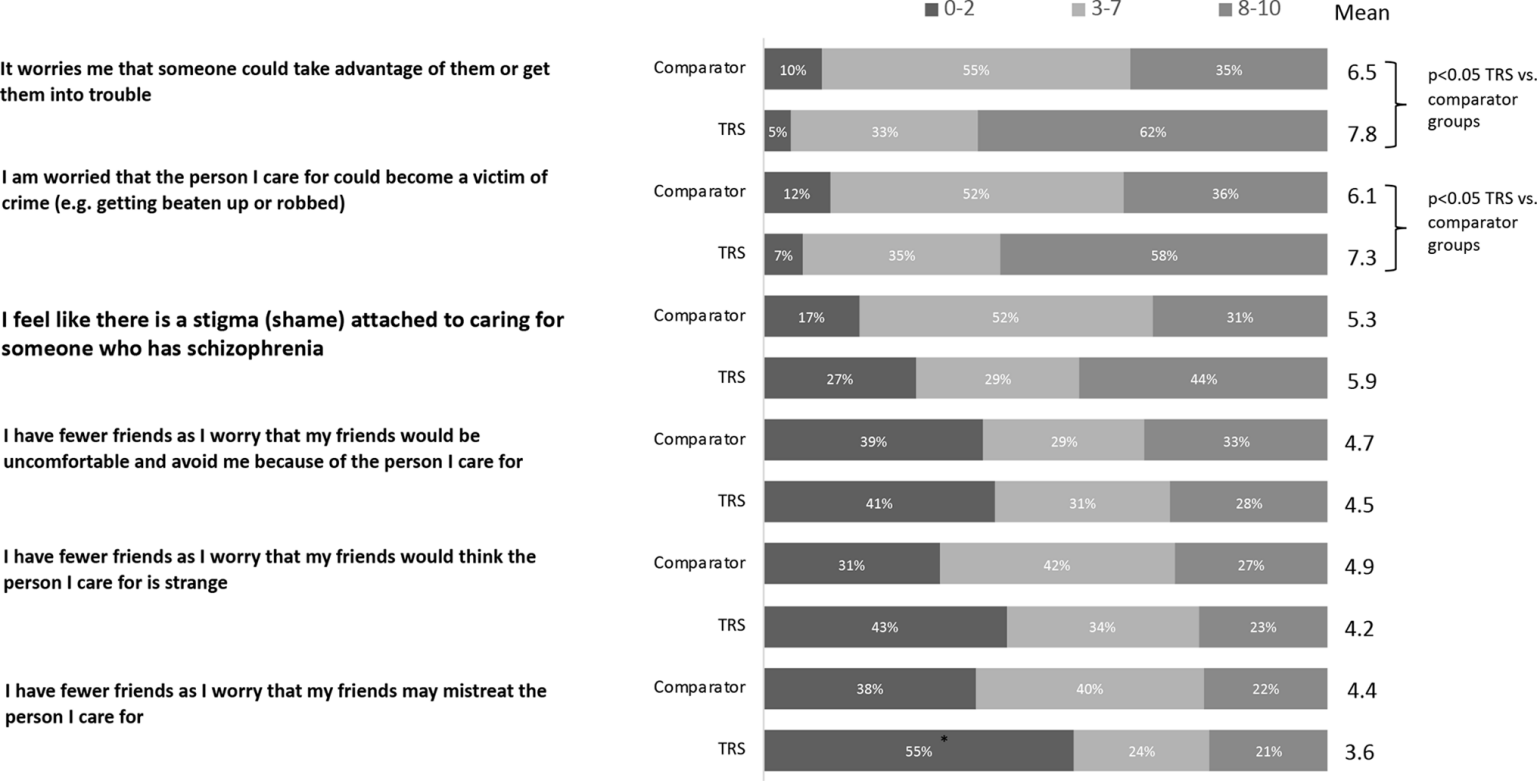
Types of emotional care provided by caregiver.

Results supported previous qualitative research confirming that majority of caregivers of people living with TRS have to provide wide-ranging emotional support. With the exception of providing “encouragement to socialize” (equally high in both groups), caregivers in the TRS group reported a higher provision of emotional care than those looking after people whose positive symptoms respond to treatment (**Figure 4**).

### Social Impact and Caregiver Health

Caregivers in both groups reported a significant impact of caring on their social life and health and reported a wide range of their own mental and physical health concerns. At the overall level, there were no significant differences between groups on social life or health. However, caregivers of people living with TRS were more likely than caregivers in the comparator group to be experiencing stress (76% *vs*. 53%, p < 0.05) and anxiety (58% *vs*. 43%, p = 0.002) and were also more likely to report a current physical health issue, with 83% of TRS caregivers reporting ≥1 physical health problem *versus* 65% of caregivers in the comparator group (p < 0.05) (**Table 3**). Conversely, caregivers in the TRS group were less likely to be experiencing obsessive thoughts than the control group (6% *vs*. 16%, p < 0.05).

**Table 3:** Impact on caregiver mental and physical health (incidence ≥5%).

	TRS group (n = 100)	Comparator group (n = 77)
**Caregiver mental health; n (%)**		
Feeling stressed	76 (76)*	41 (53)
Anxiety	58 (58)*	33 (43)
Feelings of guilt	47 (47)	26 (34)
Insomnia	42 (42)	26 (34)
Depression	41 (41)	24 (31)
Problems staying focused	28 (28)	15 (20)
Poor short-term memory	19 (19)	21 (27)
Obsessive thoughts	6 (6)*	12 (16)
Alcohol abuse/dependence	2 (2)	7 (9)
None	7 (7)	5 (7)
**Caregiver physical health; n (%)**		
High blood pressure	27 (27)	14 (18)
Obesity	21 (21)	13 (17)
Chronic fatigue	20 (20)	12 (16)
High cholesterol	20 (20)	6 (8)
Chronic pain	19 (19)	11 (14)
Migraines	17 (17)	15 (20)
Smoking	16 (16)	10 (13)
Irritable bowel syndrome (IBS)	13 (13)	5 (7)
Thyroid dysfunction	13 (13)	3 (4)
Diabetes	10 (10)	2 (2.6)
Eczema	10 (10)	2 (3)
Sleep apnea	9 (9)	5 (7)
Other type of rashes	7 (7)	3 (4)
Any other physical health condition(s)	4 (4)	5 (7)
None	17 (17)	27 (35)

*p < 0.05 TRS vs. comparator group.

### Financial Support

Overall, more caregivers in the TRS group reported essential, every day involvement in managing finances than the comparator group (50% *vs*. 33%, respectively; p < 0.05). A further 26% of caregivers in the TRS group reported “significant” involvement (a few times per week) compared with 46% in the comparator group (p < 0.05). Rates of limited/no involvement were more similar between the two groups (24% and 22%, respectively).

While more caregivers in the comparator group reported paying for out-of-pocket expenses (74% in the TRS group *vs*. 86% in the comparator group), it is relevant to note that TRS caregivers reported a non-significant trend to a greater out of pocket average ($490 *vs*. $373, respectively). The range of payments made was large in both groups: from $10 to $3,500 in the TRS group, and from $5 to $2,500 in the comparator group. Moreover, 60% of caregivers in the TRS group reported having no financial assistance compared to 37% in the comparator group. Sources of help included governmental and charitable and family/friends. Conversely, of the 40% of TRS caregivers who did receive assistance, over half (56%) received Supplemental Security Income (SSI) support compared with just 12% of caregivers in the comparator group. SSI support for the care recipient was also more common in the TRS *versus* comparator groups (44% *vs*. 19%, p < 0.05).

### Stigmatization

**Figure 5:**
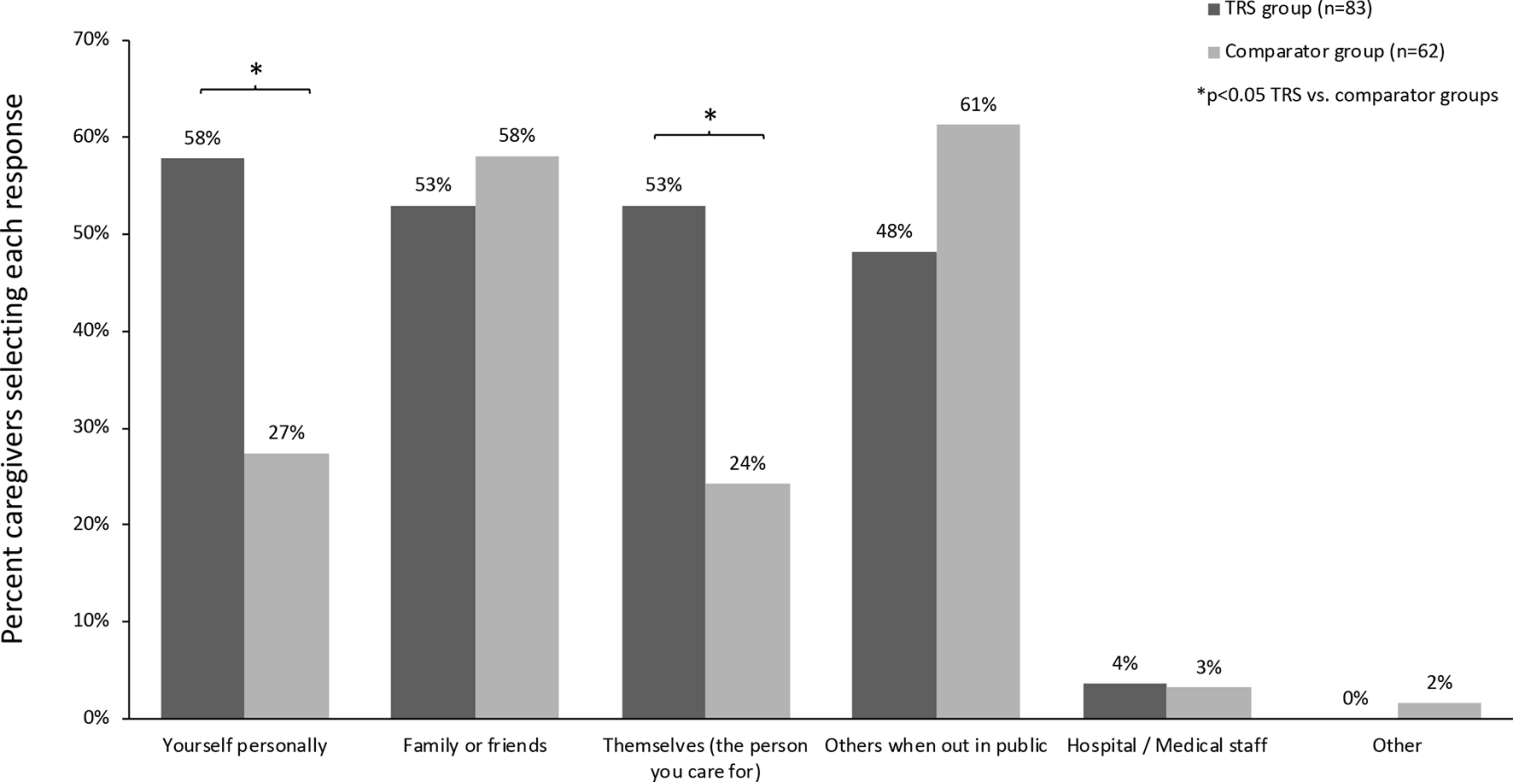
Agreement with statements related to stigma (% of caregivers rating on a 0–10 scale).

Caregivers were asked to rate their level of agreement (scale 1–10) with a range of statements related to stigma. When asked to rate the extent that the care recipient is stigmatized due to schizophrenia, caregivers in the TRS group rated stigma burden higher than in the comparator group (score of 7.8 *vs*. 6.6, p < 0.05). Further analysis showed that caregivers of people living with TRS are most concerned with the impact upon the care recipient (rather than the effect on themselves) (**Figure 5**).

### Fears for the Person Living With Schizophrenia

**Figure 6:**
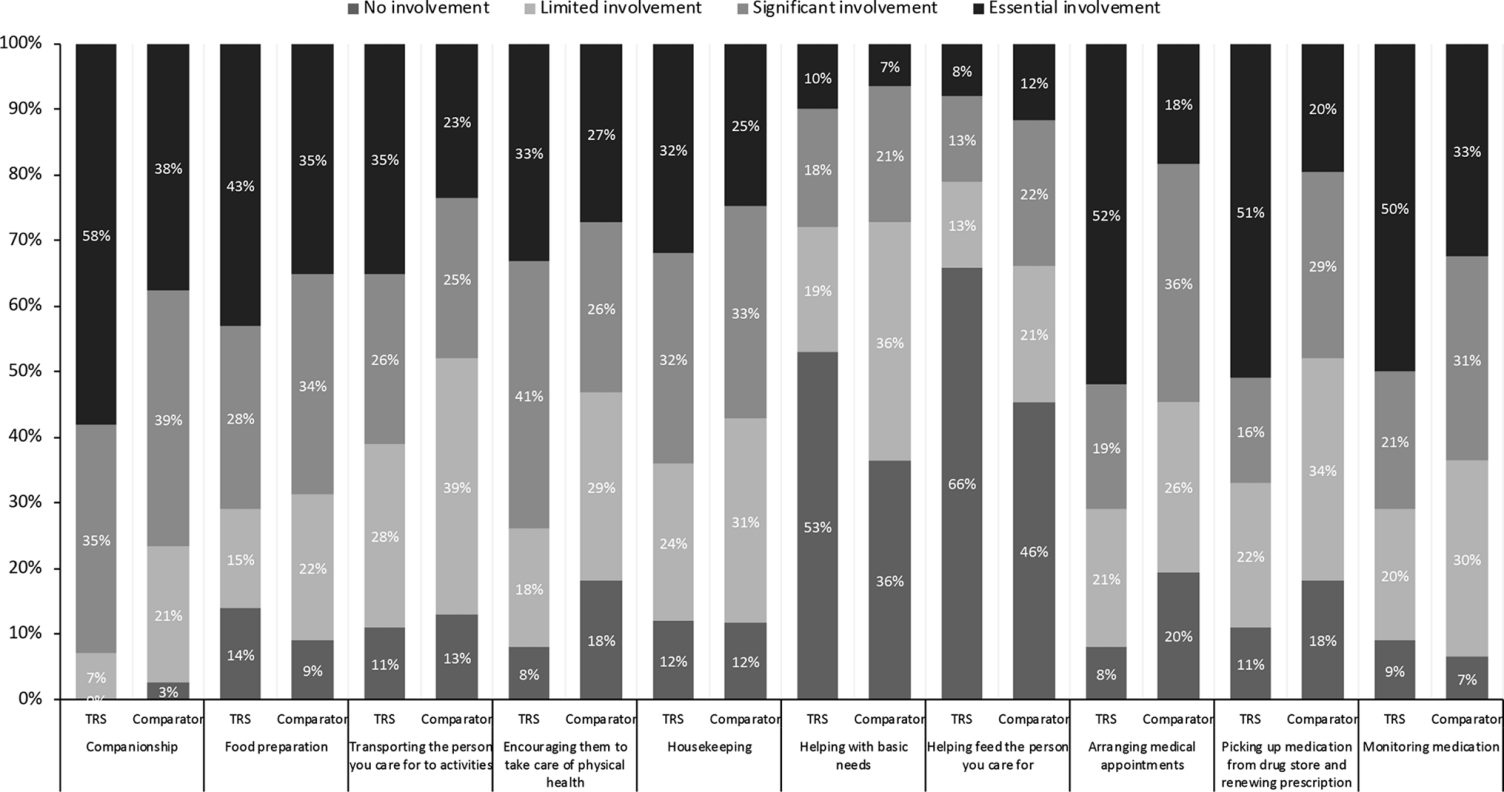
Fears of the care recipient causing harm to self or others.

Over half of caregivers looking after a person living with TRS feared that the care recipient could cause harm to: the caregiver themselves (58%), family or friends (53%), or cause self-harm (53%). Fears of harm against the caregiver themselves (58% *vs*. 27%, p < 0.05) and self-harm (53% *vs*. 24%, p < 0.05) were more prevalent in the TRS *versus* comparator group (**Figure 6**). Further questioning of the TRS group found that symptoms of agitation/hostility (identified by 68% of caregiver), delusions (60%), suspiciousness/persecution (59%), and hallucinations (45%) were key drivers of fear of causing harm.

Across both the TRS and comparator groups, the presence of agitation (likelihood score of 7.55 *vs*. 6.29 out of 10), delusions (7.22 *vs*. 6.09), and hallucinations (7.02 *vs*. 5.27) were consistently rated as the top three symptoms likely to cause the care recipient to be hospitalized.

## Discussion

Recent qualitative work suggested a high burden of caring for people living with TRS (5). This study takes the next step by quantifying the wide-ranging burdens of care for TRS and by comparing this burden with that experienced by caregivers of people whose positive symptoms of schizophrenia respond to medication. We confirm that caring for a person living with TRS places considerable burden on caregivers’ lives, with the presence of persistent psychotic symptoms (and especially positive symptoms) driving further burden, as highlighted by a number of quantitative differences between the TRS and comparator groups.

Caregivers of both groups reported high levels of “essential” involvement in a broad range of daily tasks, with higher levels of direct and indirect involvement by the TRS group. Overall, caregivers of persons living with TRS spent a mean ± SD of 61 h providing direct care *versus* 40 h for the comparator group. In both cases, this is higher than the mean of 22–23 h reported by caregivers in other surveys (19, 20). Of particular note, the vast majority (96%) of caregivers of people living with TRS reported being on call “24/7” and for a mean of 163 (of a total 168) h per week. From the economic perspective, TRS caregivers reported a greater monthly out of pocket average ($490 *vs*. $373, respectively), although there was significant variation in the amount spent per month (from $10 to $3,500). While we did not ask caregivers to extrapolate the economic costs in terms of lost income caused by caregiving *versus* employed work, one can imagine a huge impact. For example, the provision of 61 h of care equates to more than a full time job. However, since we did not collect information on the caregiver’s own relationship status (unless they were the care recipient’s spouse), the occupation/income of any partners, and other potentially important socioeconomic factors, these economic data should be viewed with caution.

As highlighted by other studies (5, 22, 23), caregivers of people living with schizophrenia (both groups) were closely involved in most aspects of daily life, including assistance with basic tasks, housekeeping, and companionship. The emotional burden of providing support was considerable, with caregivers of people with TRS reporting greater impact than those in the comparator group. As suggested in the earlier qualitative study (5), caregivers of people living with TRS were also more likely to be experiencing stress or anxiety themselves and were also significantly more likely to report at least one physical health issue. As in the qualitative study (5), the majority (93% in both groups) of caregivers reported impact on their mental health supporting the idea that the mental/emotional strain of caring for a person with schizophrenia may be one of the most significant impacts of providing care. In our study, the rates of comorbidities for caregivers in the comparator group were in line with a previous study of health outcomes for schizophrenia caregivers (type not defined) and are considerably higher than that reported for people not in a caregiver role (28).

Apart from the illness itself, the stigma surrounding schizophrenia places a major burden on patients and their caregivers. Among all mental health conditions, with the exception of substance use disorders, people living with schizophrenia face the strongest public rejection, with perceptions of unpredictability and danger (29). While much progress in public mental health literacy has been made in recent years, attitudes to people living with schizophrenia have not improved (30). It is therefore concerning that caregivers in the TRS group rated stigma higher than those in the comparator group, highlighting the need for improved care for the most functionally disabled. Stigma also leads to social distancing, which is likely to be a key contributor to the high reliance of care recipients with TRS on their caregivers—of which 62% said they were the sole caregiver. Further analysis showed that caregivers of people living with TRS acknowledge the stigma attached to their role but are mostly concerned with the impact upon the care recipient themselves.

When TRS caregivers were asked about specific symptoms and behaviors, it becomes apparent that the presence of persistent agitation/hostility, delusions, and suspiciousness/persecution are distressing and lead caregivers to worry about their own and others’ safety, as well as likelihood to cause admittance to psychiatric hospital. In line with other evidence that female family members are often the main targets of rare acts of serious violence (31), we found that 58% of TRS caregivers (of which half were parents, and most of these were mothers) feared harm to themselves. On the other hand, it is also well accepted that people living with schizophrenia are much more likely to be victims of violence than to be the originator (32, 33). In the prior qualitative survey, TRS caregivers reported fearing for their care recipients’ safety primarily because of their unpredictable behavior due to persistent positive symptoms (5), and these findings were replicated in this qualitative study. These data therefore serve to highlight the significant impact that persistent positive symptoms have on feelings of safety for both the caregivers and significant others as well as the care recipient themselves. As such, the effective management of psychotic symptoms and related behaviors should be considered a key priority in TRS.

The presence of more severe and frequent persistent positive symptoms and related behaviors was also observed to directly impact caregiver burden. In particular, when looking at the TRS group, we found that agitation/hostility and suspiciousness/persecution also were the key drivers of overall burden as assessed by the SCQ. By contrast, caregivers in the comparator group reported that poor rapport, cognitive impairment, and disorganized speech were more burdensome. Considering that many care recipients in the comparator group had previously experienced at least moderate positive symptoms, these data may indicate that caregivers of people with treatment-responsive schizophrenia become more concerned by negative and cognitive symptoms once positive symptoms are controlled. Adequate control of negative symptoms is important for community reintegration and quality of life, and it is notable that over a third of patients in the comparator group had moderate–severe negative symptoms and/or cognitive problems. While caregivers of persons living with TRS were also burdened by negative symptoms, they were perceived as less of an issue than positive symptoms. As such, our findings support the notion that the presence of persistent positive symptoms underpins the high burden related to TRS and is a root cause of the vicious cycle of other negative consequences associated with schizophrenia (8).

To the best of our knowledge, this is the first study to quantify the burdens of caring for a person living with TRS and to compare these burdens with those of caring for people whose positive symptoms of schizophrenia respond to treatment. Strengths of this study include the relatively large sample sizes which allowed for exploratory statistical comparison between groups. We conducted pilot surveys to ensure appropriate language, and descriptions were used. While the breadth of the survey can be considered a strength, it should be noted that most of the questions were not previously validated. To reflect this empirical approach, analyses were thus primarily based on T-tests without adjustment for multiplicity, although we also performed a MANOVA on the SCQ part of the survey (which has been previously validated). As such, the statistical differences in our data should be viewed as indicating areas for further systematic evaluation. Sample characteristics were generally as expected for the care recipients, with a longer time since diagnosis and higher rates of unemployment in the TRS group. However, it should be noted that caregivers were recruited to specific target quotas, and as such, we could not investigate the influence of demographic characteristics on burden. It should be emphasized that all data presented is based on caregiver report. While it is essential to understand the caregiver’s own viewpoint and perceptions of the situation, some of the data may be more open to recall bias. For example, although we made best efforts during screening to ensure that the care recipients were ≥80% adherent to their current medications, this may have been difficult for caregivers to accurately estimate. Likewise, we rely on their descriptions of symptom severity, or response to treatment, and did not cross-check our findings with any clinical data. Our survey focused on the burden of persistent positive symptoms since our previous qualitative work showed that they remain central to caregiver burden (5). As such, the sample was not selected to address the burden of persistent negative and cognitive symptoms, although as can be seen from the results, many patients in the TRS group had also poor rapport, social withdrawal, and cognitive dysfunction.

It is well accepted that not all caregivers will participate in research, and that there is some evidence that high time investment and high burden predict willingness to participate, thereby potentially biasing the results toward those with higher burden (34). A high percentage of caregivers were white Caucasian and college educated, which may limit the generalizability of the results, since caregivers often have lower prior socioeconomic status and educational level (caregiver statistics) (33, 35). It is also important to emphasize that recruitment criteria for the comparator group were not designed to be reflective of all treatment-responsive schizophrenia but rather to include patients whose positive symptoms were responsive to treatment (although not necessarily fully controlled). Patients in the comparator group could, and did, have significant persistent negative and cognitive symptoms despite an overall response to antipsychotic treatment. Moreover, we specifically targeted caregivers with significant time investment (≥20 h per week) which likely skews the comparator group to the more severe end of the treatment-responsive spectrum. Likewise, due to the need for a specialized recruitment process, we were unable to investigate non-consent bias which may have influenced our results. Finally, it should be noted that caregivers of individuals currently treated with clozapine were ineligible for this study. Prior treatment with clozapine was allowed provided that it had not been discontinued due to inefficacy (patients whose symptoms do not respond to clozapine could be considered as an “ultra” treatment-resistant subgroup). It could be argued that this patient subgroup should be included in the TRS group for inclusivity, and it could also be argued that inclusion of previous treatment with clozapine in the comparator group might mean that some of the patients recruited had TRS (again potentially minimizing differences between the two groups). As can be seen in our own results, many patients with TRS discontinue (or never try) clozapine due to the need for close monitoring/blood draws or side effects, leaving a large subset of patients with TRS without hope of an effective treatment. This survey therefore highlights the urgent unmet need for novel agents that effectively treat TRS.

## Conclusion

The results of this survey paint a vivid picture of the considerable burdens of caring for people with schizophrenia. Our findings of high caregiver burden across both groups emphasize the need for strong caregiver support and highlight some of the added burdens and stress points for those caring for people with TRS. When antipsychotic medications are ineffective for positive symptoms, caregivers face a burden that impacts all aspects of their life. Caregivers of people with TRS may benefit from tailored coping and distress tolerance interventions that specifically consider aspects of coping with persistent agitation/hostility, delusions, and suspiciousness/ persecution.

## Data Availability

Data are available upon reasonable request to the corresponding author.

## Ethics Statement

Ethical review and approval was not required for the study on human participants in accordance with the local legislation and institutional requirements. The patients/participants provided their written informed consent to participate in this study. No animal studies are presented in this manuscript. No potentially identifiable human images or data is presented in this study.

## Author Contributions

All authors conceptualized the survey and contributed to the interpretation of results and to the final version of the article. CB initiated the study, and CB and L–BD secured the funding. D–IV, CB, L–BD and OA interpreted the data, were involved in writing the publication, and read and approved the final manuscript.

## Conflict of Interest Statement

DV is a consultant for H. Lundbeck A/S and for Otsuka Pharmaceuticals, Inc.; has received research grants from Alkermes plc and Boehringer Ingelheim Pharmaceuticals, Inc.; and has served as a speaker for Janssen Pharmaceutical Companies and Otsuka Pharmaceuticals, Inc. CB and LBD are employees of H. Lundbeck A/S. OA is a consultant for Janssen-Ortho (Johnson & Johnson); Otsuka; Lundbeck; Sumitomo Dainippon Pharma and Minerva Neurosciences Inc, has received research grants from Janssen-Ortho, Otsuka, Boehringer Ingelheim, Neurocrine Bioscience, Acadia, Syneurx and diaMentis, and has served as a speaker for Janssen-Ortho, Lundbeck, Mylan Pharmaceuticals; Otsuka, HLS Therapeutics and Novartis.

This work was funded by H. Lundbeck A/S. H. Lundbeck A/S participated in designing the questionnaire and study, in analysis and interpretation of the data, and in review, approval of, and decision to submit the manuscript.
